# The Impact of the COVID-19 Pandemic on Young Adults’ Mental Health in Switzerland: A Longitudinal Cohort Study from 2018 to 2021

**DOI:** 10.3390/ijerph20032598

**Published:** 2023-01-31

**Authors:** Simon Foster, Natalia Estévez-Lamorte, Susanne Walitza, Meichun Mohler-Kuo

**Affiliations:** 1 Department of Child and Adolescent Psychiatry and Psychotherapy, Psychiatric University Hospital Zurich, University of Zurich, 8032 Zurich, Switzerland; 2 La Source, School of Nursing Sciences, HES-SO University of Applied Sciences and Arts of Western Switzerland, 1004 Lausanne, Switzerland

**Keywords:** COVID-19, depression, anxiety, attention-deficit/hyperactivity disorder, alcohol drinking, young adults

## Abstract

Most of the studies that examine the effect of the COVID-19 pandemic on mental health have been restricted to pandemic mental health data alone. The aim of the current study was to estimate the pandemic’s effect on young Swiss adults’ mental health by comparing pandemic to pre-pandemic mental health. Longitudinal data of 1175 young Swiss adults who participated in the S-YESMH study in 2018 and were followed-up in 2020 and 2021 were analyzed. The study outcomes were self-reported symptoms of depression, generalized anxiety disorder (GAD), attention-deficit/hyperactivity disorder (ADHD), thoughts about death or self-harm, and risky single-occasion drinking (RSOD). Generalized estimation equations, logistic regression and statistical mediation analysis were used to analyze the data. Evidence was found of increased depression, GAD, and ADHD among young women and increased depression among young men, resulting from the COVID-19 pandemic. Uncertainty about the future predicted young women’s depression and anxiety in 2021. COVID-19 stress in 2021 fully mediated the effect of COVID-19 stress in 2020 on depression and GAD in 2021. Young Swiss women’s and men’s mental health appears to have been adversely affected by the COVID-19 pandemic, especially during the second pandemic year. Uncertainty about the future and stress becoming chronic in 2021 likely explain some of the adverse effects.

## 1. Introduction

The COVID-19 pandemic has posed challenges to the psychological well-being of the general population, due to the numerous difficulties associated with social disruption, social isolation, financial insecurity, confinement-related stress and uncertainty [1,2,3,4]. Young adults (i.e., individuals aged approximately 19–30) could be especially vulnerable to such social disruptions [5,6] because young adulthood is a critical developmental stage that involves substantial changes in all life domains [7,8] and in which psychiatric disorders often present [9,10]. Disruptions caused by the pandemic that affect multiple aspects of life contemporaneously may make it more difficult for young adults to cope with these developmental challenges, making them more vulnerable to adverse mental health outcomes. Indeed, young adults reported worse mental health outcomes than other age groups during the COVID-19 pandemic [11,12].

However, there have been distinctive challenges to assessing the pandemic’s effect on mental health. Specifically, since the general human population has been affected, there is no unexposed control group [5]. The only control condition that might be available is the time before the pandemic, allowing us to compare people to themselves before versus during the pandemic. Studies that use such a design to investigate the impact of the COVID-19 pandemic on young adults reported evidence of a detrimental pandemic effect on depression symptoms [2,13,14,15,16], anxiety symptoms [5,13,14], and general mental distress [6,17,18], whereas one study found a reduction in internalizing symptoms [19], and one study found also a reduction in alcohol consumption [2]. However, most of the studies used convenience samples or community samples from only a particular region of a country [2,5,13,14,15,19]. Only a few studies have used nationally representative population-based samples [6,16,17,18] (for a review, see [3]). Furthermore, studies that compare mental health status before versus during the pandemic have mainly focused on the changes that occurred during the first lock-down and/or the few months that followed. Less research has been conducted on the later stages of the pandemic [14]. However, the prolonged exposure to the disruptions caused by the pandemic have likely exacerbated the difficulties and uncertainties experienced by young adults and, thus, may have further increased their likelihood of developing mental health problems.

In Switzerland, few studies have used pre-pandemic data to assess the impact of the COVID-19 pandemic on mental health [13,19]. Only one used general population-representative data. However, for this study, researchers addressed emotional distress in young adults during the first lock-down and used a sample from one city (Zurich) [19]. To our knowledge, no national data on young adults have been published that cover all of Switzerland and include the later pandemic stages. To address these gaps, we examined the impact of the COVID-19 pandemic on several indicators of mental health in a national sample of young Swiss adults in both the first and second pandemic year. To do so, we used a sample from the Swiss Youth Epidemiological Study on Mental Health (S-YESMH), a nationally representative study of young Swiss adults who were initially surveyed in 2018 and re-surveyed again during the pandemic in 2020 and 2021. Comparing pandemic and pre-pandemic mental health using within-subject analysis allowed us to estimate the pandemic’s effect in both the first and second pandemic year. In addition, we explored the impact of COVID-19-related stress on depression and anxiety, while controlling for pre-pandemic covariates.

## 2. Materials and Methods

### 2.1. Study Design and Sample

We used data from the S-YESMH cohort (see [20] for details), a longitudinal cohort of young Swiss adults who were initially surveyed in 2018. S-YESMH recruited a random national sample of young adults legally residing in Switzerland and born between 1996 and 2000. Data were collected via an online survey from February to August 2018. A total of 3840 participants (response rate = 41.4%) completed the 1st survey in 2018.

In 2020, we conducted the 1st follow-up survey. A letter of invitation, followed by two reminders, was sent to each 2018 survey participant from July to September 2020. By mid-October 2020, 1627 young adults had participated (response rate = 44.5%) [1]. The survey was adapted to include stress perceived to be related to the COVID-19 pandemic. In 2021, we conducted a 2nd follow-up survey using the same procedure. A letter of invitation with a CHF 20 gift card from a supermarket was sent out between the end of June and mid-August 2021 and 1175 responded to the 2nd follow-up (response rate = 72.2%).

### 2.2. Mental Health Outcomes

We examined six mental health outcomes. First, self-reported symptoms of current depression were assessed using the Depression module of the Patient Health Questionnaire (PHQ-9), a validated instrument used for screening individuals with probable major depression [21]. The PHQ-9 asks for the presence of the nine DSM-IV depression symptoms over the past two weeks. “Cases” were defined as respondents with a total score of at least 10 [21], indicating at least moderately severe depression. Second, item 9 on the PHQ-9 was dichotomized (“not at all” vs. at least “several days”) and used as an indicator of thoughts of death or self-harm. Third, symptoms of generalized anxiety disorder (GAD) were assessed via the GAD-7, a validated seven-item scale for assessing probable GAD [22], which asks about the presence of seven GAD symptoms over the past two weeks. Cases were defined as respondents having a total score of at least 10 [21], indicating at least moderately severe anxiety. Fourth, self-reported symptoms of attention-deficit/hyperactivity disorder (ADHD) were assessed via the Adult ADHD Self-Report Scale Screener (ASRS-v1.1), which is a validated six-item instrument used for identifying probable ADHD during the past six months [23]. Cases were defined as respondents having a total score of at least 14 [1]. Finally, risky single-occasion drinking (RSOD) was defined as consuming at least six standard drinks on a single occasion for men and four standard drinks for women, as in previous studies [20]. The definition of a standard drink was clarified using pictures with examples in the questionnaire. Participants were asked for the frequency with which they engaged in RSOD. The following two RSOD variables were created: RSOD at least monthly (“no”/“yes”) and RSOD weekly (“no”/“yes”).

One must note that although the measures we used for depression, GAD and ADHD can be used to screen for probable cases, they do not provide sufficient information to establish a formal clinical diagnosis. Therefore, in the context of our study, the terms “depression”, “GAD”, and “ADHD” refer to people who reported corresponding symptoms and probably suffer from the corresponding disorder.

### 2.3. COVID-19-Related Stressors

Perceived COVID-19-related stress in both 2020 and 2021 was measured using the first part of the Responses to Stress Questionnaire (RSQ COVID-19) developed by the Stress and Coping Research Lab at Vanderbilt University [24]. It includes a list of 14 situations caused by COVID-19 that respondents sometimes find stressful or have a problem dealing with. Respondents rate each specific situation in terms of how stressful each stressor was using a four-point scale (from 0 = “not at all” to 3 = “very”). We reported the percentage of participants who answered “somewhat” or “very” and created an index of total stress by averaging the 14 items (range: 0–3).

### 2.4. Other Covariates

One potential major confounder of the comparison between pre-pandemic and pandemic mental health status was the participant’s age (in years). If developmental trends for mental health status were expected to occur within the study participants’ age range (17.1–22.3 years in 2018) [9,10], changes from pre-pandemic to pandemic mental health status could be due to such normative developmental processes rather than the pandemic itself. We, therefore, included age as a time-varying covariate in the within-subject comparisons of pre-pandemic and pandemic mental health status.

We additionally included several pre-pandemic variables to account for different baseline risks. First, two indicators of the participants’ pre-pandemic economic situation were included. Participants were asked to estimate their approximate monthly household income (answer options “less than 6000 Swiss francs (CHF)”/“about CHF 6000”/“more than CHF 6000”/“don’t know”/“don’t want to specify”) and whether they are able to always pay all their bills without any problems (“yes”/“no”/“don’t know”) [20]. Second, the participants’ pre-pandemic citizen status (Swiss; non-Swiss) in 2018 was provided by the Swiss Federal Statistical Office [20]. Third, the participants’ language region of origin was provided by the Swiss Federal Statistical Office [20]. Fourth, for each of the six mental health indicators listed above, the pre-pandemic status of the remaining mental health indicators were included as covariates. Monthly and weekly RSOD were collapsed into a single, three-category variable for this purpose (“less than monthly”/“monthly”/“weekly”). When modelling depression as an outcome, the “thoughts of death or self-harm” indicator was excluded, because it is an element within the depression measure. Finally, sex, as provided by the Swiss Federal Statistical Office, was accounted for by means of stratified analyses, as explained in the statistical analysis section.

### 2.5. Statistical Analysis

Because a previous study that investigated this study cohort found sex differences in mental health [1], all analyses performed were sex-stratified. Within-subject comparisons of pre-pandemic and pandemic mental health were performed using generalized estimation equations (GEE) for binary outcomes and an unstructured working correlation matrix, in order to deal with the measurements being clustered within individuals [25]. Time was modelled as a factor, with 2018 set as the reference level against which the years 2020 and 2021 were compared. An unadjusted model and a model that included time-varying age and pre-pandemic covariates, as outlined in the covariate-section, were tested.

GEEs for binary outcomes were used to examine the changes in the respondents’ experiences with each of the 14 COVID-19-related stressors from 2020 to 2021. For each stressor, a model with categorical time (2020 vs. 2021) and an exchangeable working correlation matrix was evaluated.

Logistic regression analyses were used to estimate the relationships between COVID-19-related stress and both depression and GAD in 2021. Unadjusted and adjusted relationships were examined (adjusting for pre-pandemic depression, GAD, age, household income, ability to pay one’s bills, citizen status, and language region).

Finally, mediation analysis was performed using the algorithm introduced by Imai et al. [26] to examine whether the relationship between COVID-19-related stress in 2020 and depression and GAD in 2021 was statistically mediated by COVID-19-related stress in 2021. The presence of such mediation would imply that higher stress in 2020 translated into higher stress in 2021, which in turn increased depression and GAD, suggesting that COVID-related stress had become chronic in 2021. Mediation effects were adjusted for pre-pandemic depression, GAD, age, household income, ability to pay one’s bills, citizen status, and language region. They were estimated as absolute risk differences between low and high COVID-19-related stress in 2020, which we defined as the 20th and the 80th quantiles of the total stress score. Non-parametric bootstrapping with 1500 bootstrap samples was used to obtain confidence intervals and *p* values.

All analyses were conducted using R software version 4.1.0 [27]. The add-on packages “geepack” [28] and “mediation” [26] were used for estimating GEEs and mediation analysis, respectively.

## 3. Results

The analytic sample included 1175 young adults who participated in all three waves of the survey, among whom 61.4% were female. In 2018, the average age was 19.6 years (±1.4 standard deviation); 85.3% had Swiss nationality; 66% were German-speaking, 27.2% French-speaking, and 6.8% Italian-speaking; the household income of 30.3% was less than CHF 6000, while the income of 42.1% was CHF 6000 or more and the remaining subjects indicated that they did not know their household income (18%) or were unwilling to disclose it (9.7%); and 10.4% indicated that they were not always able to pay their bills. In addition, 78.6% were still enrolled in education in 2018; in 2020, 4.1% indicated obligatory school as their highest achieved education, 78.2% indicated secondary vocational or higher school education, 15% indicated tertiary education, and 2.7% some other education. The participants’ 2018 characteristics that predicted drop out status (defined as participating in all three waves vs. only in the first wave) were weekly RSOD, being male, not having Swiss citizen status, having a household income less than CHF 6000 not being able to pay one’s bills by the end of the month, and being German- or French-speaking citizens (vs. Italian-speaking, see Table S1 in the online Supplementary Materials).

**Figure 1:**
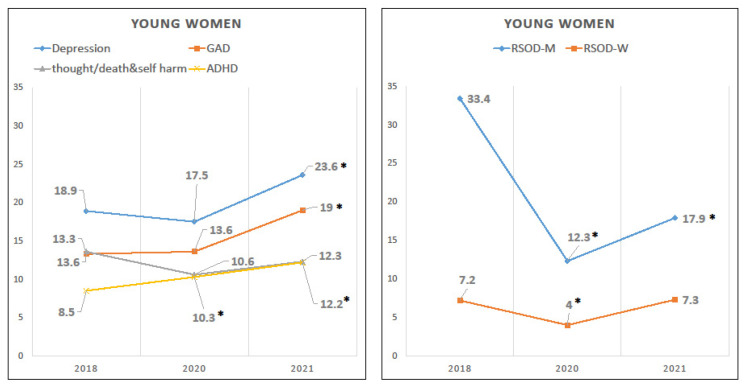
Unadjusted prevalence rates of young women’s mental health problems from 2018 to 2021. * *p* ≤ 0.05 (vs. reference year 2018) in adjusted model 2. GAD = Generalized anxiety disorder. ADHD = Attention-deficit/hyperactivity disorder. RSOD-M = Risky single-occasion drinking at least monthly. RSOD-W = Risky single-occasion drinking at least weekly.

Young women’s prevalence of depression and GAD remained stable from 2018 to 2020, but increased in 2021 (Table 1, Figure 1). Specifically, the depression rate increased from 18.9% to 23.6% and this increase remained statistically significant in the adjusted model (OR (95% CI) = 1.72 (1.17, 2.54), *p* = 0.0061). GAD prevalence increased from 13.3% to 19.0% and this increase also remained statistically significant in the adjusted model (OR (95% CI) = 1.59 (1.02, 2.47), *p* = 0.0425). The prevalence of ADHD increased gradually from 8.5% (2018) to 10.3% (2020) to 12.2% (2021). Both increases were statistically significant in the adjusted model (OR (95% CI) = 1.53 (1.03, 2.29), *p* = 0.0352; OR (95% CI) = 2.10 (1.29, 3.40), *p* = 0.0027).

On the other hand, the prevalence of monthly RSOD declined to 12.3% in 2020 and was still just 17.9% in 2021, compared to 33.4% in 2018. These reductions remained statistically significant in the adjusted model (OR (95% CI) = 0.23 (0.17, 0.31), *p* < 0.0001; OR (95% CI) = 0.34 (0.24, 0.48), *p* < 0.0001). The prevalence of weekly RSOD dropped in 2020, relative to 2018, from 7.2% to 4.0% (adjusted OR (95% CI) = 0.41 (0.25, 0.68), *p* = 0.0005), but was restored to the 2018 level in 2021. Finally, there was no statistical evidence of change in their thoughts about self-harm or death.

**Figure 2:**
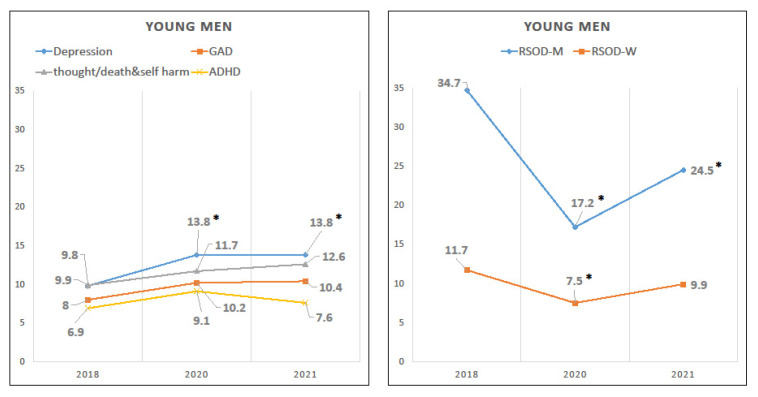
Unadjusted prevalence rates of young men’s mental health problems from 2018 to 2021. * *p* ≤ 0.05 (vs. reference year 2018) in adjusted model 2. GAD = Generalized anxiety disorder. ADHD = Attention-deficit/hyperactivity disorder. RSOD-M = Risky single-occasion drinking at least monthly. RSOD-W = Risky single-occasion drinking at least weekly.

Young men’s prevalence of depression increased from 9.8% in 2018 to 13.8% in 2020 and remained at an increased level in 2021 (Table 2, Figure 2). Prevalence rates for 2020 and 2021 both differed significantly from 2018 in the adjusted model (OR (95% CI) = 2.05 (1.27, 3.30), *p* = 0.0032; OR (95% CI) = 2.37 (1.32, 4.23), *p* = 0.0036).

Conversely, the prevalence of monthly RSOD reduced to 17.2% in 2020 and was still only 24.5% in 2021, versus 34.7% in 2018. These reductions remained statistically significant in the adjusted model (OR (95% CI) = 0.35 (0.25, 0.49), *p* < 0.0001; OR (95% CI) = 0.51 (0.35, 0.75), *p* = 0.0007). The prevalence of weekly RSOD fell from 11.7% in 2018 to 7.5% in 2020 (adjusted OR (95% CI) = 0.50 (0.32, 0.77), *p* = 0.0018), but in 2021, it was no longer statistically different than the 2018 rate. There was no statistical evidence of change in GAD, thoughts of self-harm or death, or ADHD.

Table 3 shows the prevalence of 14 COVID-19-related stressors. Descriptively, the prevalence rates were consistently higher among women than men. In line with this, the women’s average total stress index was higher than the men’s average in 2020 (0.98 vs. 0.73, *p* < 0.001) and in 2021 (0.95 vs. 0.66, *p* < 0.001). Among women, two of the most prevalent stressors increased from 2020 to 2021 (stressors 3 and 4), while six stressors decreased (stressors 2, 6, 7, 8, 9 and 13) and the remaining six remained approximately stable. Importantly, considering the stressors with the highest prevalence rates across the years (i.e., prevalence rates higher 0.40), one remained stable (“Not being sure about when COVID-19 will end or what will happen in the future”) and two increased (“Unable to participate in social activities and normal routines”; “Having to change, postpone, or cancel important plans or events”), while only one decreased from 2020 to 2021 (“Unable to spend time in person with my friends or family”). Among young men, four stressors decreased from 2020 to 2021 (2, 6, 8, 9), whereas the remaining 10 stressors remained approximately stable. One of the decreasing stressors was “Unable to spend time in person with my friends or family”, the stressor that had the highest prevalence rate in 2020.

Table 4 shows the results of three major stressors in 2021 that predicted depression and GAD. In the unadjusted analyses, all three stressors were significantly associated with depression and GAD. When mutually adjusted, uncertainty about the future (“Not being sure about when COVID-19 will end or what will happen in the future”) was a significant predictor of depression and GAD in both women and men, whereas having to change, postpone, or cancel important plans or events also was associated with women’s depression. When adjusting additionally for pre-pandemic covariates, there was no longer statistical evidence of a relationship between any stressor and men’s depression or GAD. In contrast, the relationships remained among women, meaning that uncertainty about the future predicted both women’s depression and GAD; and having to change, postpone, or cancel important plans and events was linked to women’s depression.

Table 5 (upper part) shows that both young women and men who experienced more COVID-19-related stress in 2020 had higher odds of depression and GAD in 2021, even after adjusting for pre-pandemic covariates. Once the COVID-19-related stress of 2021 was considered, these relationships were substantially reduced and no longer statistically significant, suggesting that the COVID-19-related stress of 2021 mediated the effect of the COVID-19-related stress of 2020. Indeed, the mediation analysis indicated that the relationships between COVID-19-related stress in 2020 and both depression and GAD were fully mediated by COVID-19-related stress in 2021 (lower part of Table 5), as indicated by the significant total and mediated effects and non-significant direct effects in both women and men.

## 4. Discussion

The present study examined the impact of the COVID-19 pandemic on mental health in a national sample of young Swiss adults by comparing mental health in the first and second pandemic year against pre-pandemic mental health, while controlling for time-varying age and pre-pandemic covariates. In addition, COVID-19-related stressors were explored as predictors of depression and GAD.

To start with, there was no evidence that the observed increases in mental health outcomes from the pre-pandemic to pandemic years could be attributed to normative age-related trends. This finding rules out a major potential confounder in this within-subject analysis, for which we treated young adults’ pre-pandemic status as an unexposed control condition, so we could estimate the pandemic’s impact on mental health.

Young Swiss women’s depression and GAD did not increase after the first pandemic wave in 2020, relative to pre-pandemic levels. The lack of increase in the summer of 2020 is consistent with a recent meta-analysis, which indicated that depression and GAD levels returned to pre-pandemic levels by the summer of 2020, after an initial increase early in 2020 during the first pandemic wave ([3], see also [17,18,29,30] for additional meta-analyses that report similar findings). The authors interpreted this discovery as evidence of an acute stress response, followed by psychological adaptation and relief. Our findings are consistent with the second half of this conjecture.

However, the situation changed in the second pandemic year when young women’s depression and GAD increased. Although we observed an increase in depression and GAD symptoms among young women, more COVID-19-related stresses decreased (six) than increased (two) significantly from 2020 to 2021, with the exception being “Unable to participate in social activities and normal routines” and “Having to change, postpone, or cancel important plans or events”, both of which increased significantly in 2021. In addition, uncertainty (“Not knowing when the pandemic will end”) remained the most commonly perceived stress among young women and the strongest predictor of depression and GAD in 2021. One possible explanation could be that the sense of uncertainty became more chronic after one year, and young women are more vulnerable to uncertainty than men are (54% women vs. 31% men). However, more research and analyses are needed to explain the increased depression and GAD among young women. While few studies examined the pandemic effects on young adults’ mental health in the second pandemic year, the observed increase is consistent with a recent meta-analysis that shows elevated anxiety and depression symptoms years after exposure to various disasters, including epidemics [31].

A different trend was evident in the prevalence of young women’s ADHD, since its prevalence increased steadily from 2018 to 2021. The increase in 2020 might be explained by the time frame of the ADHD measure, which asked about symptoms over the past six months and, therefore, included the lockdown period for at least some women. This could explain why it had already increased in 2020, after the initial pandemic wave. Alternatively, the 2020 increase might be a substantial effect due to the loss of structure and daily routines caused by the anti-pandemic policy measures and the negative impact of ADHD symptoms on the adaptation to the COVID-19 outbreak [32,33,34]. Regardless, the increased ADHD prevalence supports the hypothesis that the pandemic had detrimental effects on mental health and is consistent with other studies that have reported such an effect on ADHD symptoms [33,35,36].

The time trend for young men’s depression differed from both the young women’s trend and the above-mentioned trend reported in the literature. Specifically, young men’s depression prevalence had already increased by the summer of 2020 and remained on this elevated level afterwards in 2021. It was thought that depression was triggered by the first pandemic wave in men, but the ongoing pandemic did not further increase their depression. Although recent meta-analyses have confirmed the large heterogeneity in mental health trajectories after the pandemic onset [3,29,30], further research is required to understand this sex difference. Regardless, the elevated level of depression symptoms in the second pandemic year is again consistent with the already mentioned meta-analysis that showed elevated depression symptoms in the long term following exposure to various disasters [31].

Young men appeared to be generally less affected by the pandemic than young women, in that they experienced no changes in GAD, thoughts of suicide or self-harm, or ADHD, relative to pre-pandemic times, in either the first or second pandemic year. Furthermore, men’s reported level of COVID-19-related stress was lower than women’s in both 2020 and 2021. Although evidence for a possibly stronger impact of the pandemic on girls’ and women’s mental health was reported before, the mechanisms are less clear. Suggested explanations include higher family and childcare responsibilities [37], higher sensitivity to interpersonal stressors [38], higher perceived loneliness and loss of social interaction [39], higher likelihood of appraising the COVID-19-related lockdown as a threat or harm and loss [40], or more worrying about pandemic-related health concerns (such as contracting or spreading the virus) [41,42]. Future research should clarify the underlying mechanisms.

Young women and men who were more stressed in 2020 were also more stressed in 2021 and this, in turn, increased their risk of depression and GAD in 2021. This provides evidence that COVID-19-related stress became chronic in 2021. The chronification of stress possibly explains why depression and GAD increased in 2021 among women and stayed on this increased level among men. It is thought that even if some initial adjustment after the first wave was possible, with stress becoming chronic, fewer people were able to adjust.

Finally, the reduced prevalence of monthly RSOD in both young men and women agrees with other studies that have documented reduced alcohol use among youth during the COVID-19 pandemic [2,43]. This drop in alcohol use is likely attributable to policy responses, such as the lock-down in 2020 and ongoing public life restrictions in 2021. However, our results add some refinement; as opposed to monthly RSOD, weekly RSOD was only reduced during the lockdown period in early 2020, returning to its pre-pandemic level in 2021. A likely explanation is that weekly RSOD drinkers are more habitual and determined drinkers who are more willing to overcome obstacles in the way of their drinking habit, while monthly RSOD is opportunity-driven to a larger degree, and hence more strongly influenced by alcohol availability [44,45,46].

The increased prevalence of several mental health indicators justifies large-scale screening for mental health symptoms during pandemics, with the goal of detecting those with subclinical symptoms in whom some form of preventative intervention might be indicated and those with untreated disorders who warrant treatment. Universal screening should be applied, since such screening has been shown to increase not just the identification of cases, but treatment initiation [47]. In addition, prevention programs and treatment provision must be scaled up proportionately with the increased prevalence of mental health problems. Our findings indicate that COVID-19-related stress early in the pandemic was an indicator of later stress and, hence, provides a starting point for selective (i.e., risk-based) prevention, together with other risk factors identified in previous research [5,16,19,48,49].

The results presented here must be considered, while simultaneously acknowledging certain study limitations. First, although we controlled for age-related mental health trends, additional processes that increase young adults’ mental health problems and took place in parallel with the COVID-19 pandemic cannot be ruled out. Second, only current episodes of depression, thoughts of death or self-harm and GAD were assessed. Our prevalence estimates, therefore, do not cover the already finished episodes that nevertheless took place during the study period. Third, dropouts are inevitable in any longitudinal study. Out of the 3840 youths who participated in the 2018 survey, only 1175 completed both the 2020 and 2021 surveys and were available for analysis. Baseline (i.e., 2018) characteristics that predicted study drop-out were weekly RSOD, being male, not having Swiss citizen status, having a household income less than CHF 6000, not being able to pay one’s bills by the end of the month, and being German- or French-speaking (vs. Italian-speaking), possibly restricting the generalizability of our results. In addition, the examined sample consisted mainly of White young adults; thus, the results may not apply to other countries, such as African or South American countries. Finally, our study did not take into account the likely expansion of the total number of stressors in the population due to stressors that emerged in the second pandemic year, such as increasing political divides over the appropriate way to deal with the pandemic or divides over vaccination.

## 5. Conclusions

Young Swiss women’s and men’s mental health appears to have been adversely affected by the COVID-19 pandemic, especially during the second pandemic year. Uncertainty about the future and stress becoming chronic in 2021 explain some of the adverse effects. One apparent positive impact of the pandemic has been a reduction in risky alcohol consumption.

## Figures and Tables

**Table 1:** Young women’s mental health problems from 2018 to 2021.

			Model 1 ^b^				Model 2 ^c^			
Symptoms	Year	Prevalence ^a^	OR	95% CI		*p*-Value	OR	95% CI		*p*-Value
Depression	2018 (ref)	0.189	1.00				1.00			
	2020	0.175	0.91	0.74	1.12	0.3804	1.04	0.76	1.42	0.8023
	2021	0.236	1.33	1.07	1.64	0.0086	1.72	1.17	2.54	0.0061
GAD	2018 (ref)	0.133	1.00				1.00			
	2020	0.136	1.02	0.79	1.33	0.8563	1.00	0.68	1.47	0.9991
	2021	0.190	1.53	1.19	1.96	0.0008	1.59	1.02	2.47	0.0425
Thoughts about death or self-harm	2018 (ref)	0.136	1.00				1.00			
	2020	0.106	0.76	0.59	0.98	0.0330	0.78	0.55	1.11	0.1694
	2021	0.123	0.89	0.68	1.18	0.4245	0.95	0.61	1.47	0.8066
ADHD	2018 (ref)	0.085	1.00				1.00			
	2020	0.103	1.24	0.93	1.64	0.1386	1.53	1.03	2.29	0.0352
	2021	0.122	1.50	1.13	2.00	0.0047	2.10	1.29	3.40	0.0027
Monthly RSOD	2018 (ref)	0.334	1.00				1.00			
	2020	0.123	0.28	0.22	0.36	0.0000	0.23	0.17	0.31	0.0000
	2021	0.179	0.43	0.35	0.53	0.0000	0.34	0.24	0.48	0.0000
Weekly RSOD	2018 (ref)	0.072	1.00				1.00			
	2020	0.040	0.54	0.36	0.81	0.0025	0.41	0.25	0.68	0.0005
	2021	0.073	1.02	0.72	1.45	0.9081	0.70	0.40	1.22	0.2058

ref = Reference category. OR = Odds ratio. CI = Confidence interval. GAD = Generalized anxiety disorder. ADHD = Attention-deficit/hyperactivity disorder. RSOD = Risky single-occasion drinking. ^a^ Unadjusted prevalence estimated from model 1. ^b^ Unadjusted. ^c^ Adjusted for time-varying age and pre-pandemic (2018) household income, ability to pay one’s bills, citizen status, language region, and other mental health symptoms.

**Table 2:** Young men’s mental health problems from 2018 to 2021.

			Model 1 ^b^				Model 2 ^c^			
Symptoms	Year	Prevalence ^a^	OR	95% CI		*p*-Value	OR	95% CI		*p*-Value
Depression	2018 (ref)	0.098	1.00				1.00			
	2020	0.138	1.47	1.05	2.07	0.0245	2.05	1.27	3.30	0.0032
	2021	0.138	1.47	1.05	2.07	0.0245	2.37	1.32	4.23	0.0036
GAD	2018 (ref)	0.080	1.00				1.00			
	2020	0.102	1.31	0.86	1.99	0.2046	1.39	0.76	2.55	0.2809
	2021	0.104	1.34	0.91	1.98	0.1385	1.47	0.73	2.98	0.2794
Thoughts about death or self-harm	2018 (ref)	0.099	1.00				1.00			
	2020	0.117	1.21	0.84	1.73	0.3098	1.46	0.90	2.39	0.1272
	2021	0.126	1.31	0.94	1.84	0.1152	1.76	0.98	3.15	0.0587
ADHD	2018 (ref)	0.069	1.00				1.00			
	2020	0.091	1.35	0.92	1.99	0.1233	1.40	0.82	2.38	0.2179
	2021	0.076	1.10	0.71	1.71	0.6548	1.04	0.51	2.11	0.9110
Monthly RSOD	2018 (ref)	0.347	1.00				1.00			
	2020	0.172	0.39	0.31	0.50	0.0000	0.35	0.25	0.49	0.0000
	2021	0.245	0.61	0.50	0.75	0.0000	0.51	0.35	0.75	0.0007
Weekly RSOD	2018 (ref)	0.117	1.00				1.00			
	2020	0.075	0.61	0.44	0.86	0.0047	0.50	0.32	0.77	0.0018
	2021	0.099	0.83	0.59	1.18	0.3018	0.60	0.34	1.06	0.0809

ref = Reference category. OR = Odds ratio. CI = Confidence interval. GAD = Generalized anxiety disorder. ADHD = Attention-deficit/hyperactivity disorder. RSOD = Risky single-occasion drinking. ^a^ Unadjusted prevalence estimated from model 1. ^b^ Unadjusted. ^c^ Adjusted for time-varying age and pre-pandemic (2018) household income, ability to pay one’s bills, citizen status, language region, and other mental health symptoms.

**Table 3:** Prevalence of COVID-19-related stressors in 2020 and 2021.

	Young Women			Young Men		
Prevalence of Somewhat/Very in %	2020	2021	*p*-Value ^a^	2020	2021	*p*-Value ^a^
My family has experienced financial problems	0.11	0.12	0.3657	0.06	0.07	0.6474
2. Unable to spend time in person with my friends or family	0.57	0.47	0.0000	0.42	0.33	0.0005
3. Unable to participate in social activities and normal routines	0.43	0.50	0.0002	0.34	0.38	0.1331
4. Having to change, postpone, or cancel important plans or events	0.49	0.53	0.0464	0.41	0.37	0.1689
5. Challenges at home or with others	0.21	0.22	0.6394	0.11	0.12	0.7255
6. My family has experienced trouble getting groceries or other needed supplies	0.07	0.04	0.0067	0.04	0.02	0.0249
7. Watching or hearing distressing news reports about COVID-19	0.35	0.28	0.0012	0.17	0.14	0.1584
8. Not being sure about myself or someone close to me getting COVID-19	0.35	0.28	0.0005	0.20	0.11	0.0000
9. Myself or someone close to me having symptoms or being diagnosed with COVID-19	0.36	0.30	0.0057	0.24	0.17	0.0017
10. Trouble getting medical care or mental health services	0.12	0.12	0.8527	0.07	0.06	0.1832
11. Not being sure about when COVID-19 will end or what will happen in the future	0.55	0.54	0.5978	0.34	0.31	0.2083
12. Difficulty completing my school/work responsibilities online	0.05	0.07	0.0568	0.04	0.05	0.3853
13. Unable to complete educational or work requirements	0.24	0.20	0.0252	0.14	0.13	0.6276
14. Needing to take on greater family and/or work responsibilities	0.13	0.14	0.5211	0.08	0.06	0.1806

^a^*p*-value for the change in prevalence rates from 2020 to 2021, as estimated by generalized estimation equations that predict the probability of each stressor by categorical time (2020 vs. 2021).

**Table 4:** Major COVID-19-related stressors of 2021 that predicted depression and GAD in 2021.

		Model 1 ^a^				Model 2 ^b^				Model 3 ^c^			
Sample, Outcome	COVID-19-Related Stressor 2021	OR	95% CI		*p*-Value	OR	95% CI		*p*-Value	OR	95% CI		*p*-Value
**Young women**													
Depression 2021	Social activities, routines	1.97	1.39	2.81	0.0002	1.39	0.94	2.05	0.0990	1.44	0.94	2.22	0.0959
	Cancel plans/events	2.40	1.68	3.48	0.0000	1.76	1.18	2.67	0.0065	1.82	1.16	2.89	0.0101
	Uncertainty about pandemic end/future	2.26	1.58	3.27	0.0000	1.72	1.17	2.55	0.0063	1.76	1.15	2.72	0.0105
GAD 2021	Social activities, routines	1.96	1.34	2.89	0.0006	1.49	0.98	2.29	0.0621	1.55	0.99	2.45	0.0580
	Cancel plans/events	1.86	1.27	2.76	0.0016	1.22	0.79	1.90	0.3659	1.19	0.74	1.91	0.4827
	Uncertainty about pandemic end/future	2.62	1.76	3.96	0.0000	2.20	1.44	3.42	0.0003	2.11	1.34	3.37	0.0014
**Young men**													
Depression 2021	Social activities, routines	2.58	1.51	4.45	0.0006	1.80	0.97	3.36	0.0645	1.25	0.62	2.52	0.5303
	Cancel plans/events	1.88	1.10	3.21	0.0200	1.05	0.57	1.95	0.8673	1.40	0.68	2.86	0.3563
	Uncertainty about pandemic end/future	3.07	1.79	5.28	0.0000	2.35	1.28	4.37	0.0062	1.96	1.00	3.89	0.0511
GAD 2021	Social activities, routines	2.43	1.32	4.54	0.0045	1.55	0.76	3.16	0.2262	1.20	0.54	2.68	0.6569
	Cancel plans/events	2.33	1.27	4.32	0.0067	1.46	0.72	2.95	0.2938	2.14	0.95	4.85	0.0668
	Uncertainty about pandemic end/future	2.91	1.58	5.41	0.0006	2.09	1.04	4.22	0.0376	1.95	0.90	4.24	0.0891

OR = Odds ratio. CI = Confidence interval. GAD = Generalized anxiety disorder. ^a^ Unadjusted. ^b^ Mutually adjusted. ^c^ Mutually adjusted and adjusted for pre-pandemic (2018) depression, GAD, age, household income, ability to pay one’s bills, citizen status, and language region.

**Table 5:** Results of logistic regression and mediation analysis relating COVID-19-related stress of 2020 to depression and GAD in 2021.

			Women				Men			
Analysis	Outcome	Predictor	OR	95% CI		*p*-Value	OR	95% CI		*p*-Value
Regression	Depression 2021	COVID-19 stress 2020 ^a^	2.64	1.82	3.85	0.0000	4.86	2.75	8.79	0.0000
		COVID-19 stress 2020 ^b^	2.01	1.30	3.11	0.0016	3.71	1.92	7.30	0.0001
		COVID-19 stress 2020 ^c^	0.85	0.50	1.42	0.5324	1.92	0.84	4.41	0.1211
	GAD 2021	COVID-19 stress 2020 ^a^	3.00	2.01	4.50	0.0000	4.73	2.54	9.02	0.0000
		COVID-19 stress 2020 ^b^	2.24	1.43	3.56	0.0005	3.97	1.87	8.57	0.0003
		COVID-19 stress 2020 ^c^	1.17	0.69	1.99	0.5525	1.49	0.58	3.84	0.4066
**Analysis**	**Outcome**	**Effect of COVID-19 Stress 2020**	**Estimate ^d^**	**BCa CI**		***p*-Value**	**Estimate ^d^**	**BCa CI**		***p*-Value**
Mediation	Depression 2021	Total	0.083	0.032	0.135	0.0000	0.078	0.030	0.119	0.0013
		Mediated	0.102	0.073	0.136	0.0000	0.038	0.010	0.069	0.0160
		Direct	−0.018	−0.075	0.041	0.5507	0.040	−0.011	0.087	0.1227
	GAD 2021	Total	0.085	0.037	0.137	0.0013	0.061	0.024	0.103	0.0000
		Mediated	0.069	0.045	0.097	0.0000	0.042	0.013	0.072	0.0080
		Direct	0.016	−0.045	0.071	0.6000	0.019	−0.032	0.068	0.4347

COVID-19 stress = index of total COVID-19-related stress. OR = Odds ratio. CI = Confidence interval. GAD = Generalized anxiety disorder. BCa CI = Bias-corrected and accelerated confidence interval obtained by the non-parametric bootstrap. ^a^ Unadjusted. ^b^ Adjusted for pre-pandemic (2018) depression, GAD, age, household income, ability to pay one’s bills, citizen status, and language region. ^c^ Adjusted for pre-pandemic (2018) depression, GAD, age, household income, ability to pay one’s bills, citizen status, language region, and COVID-19-related stress of 2021. ^d^ Effect estimates of mediation analysis represent absolute risk differences between low and high COVID-19 stress in 2020, defined as the 20th (low) and the 80th (high) quantiles of COVID-19 stress in 2020. The displayed effects correspond to the average effects.

## Data Availability

The datasets analyzed in the current study are not publicly available due to the conditions specified in the data protection contract for this study.

## Supplementary Materials

The following supporting information can be downloaded at: https://www.mdpi.com/article/10.3390/ijerph20032598/s1, Table S1. Odds ratios obtained from a logistic regression model that predicts drop-out status (participating in all three waves vs. only in the first wave) using 2018 study variables.
