# Supplementary material for: The Impact of the COVID-19 Pandemic on Young Adults’ Mental Health in Switzerland: A Longitudinal Cohort Study from 2018 to 2021
Source: Int J Environ Res Public Health. 2023 Jan 31;20(3):2598. doi: 10.3390/ijerph20032598 (PMC9915161; doi:10.3390/ijerph20032598)
Supplement: Supplementary file 1 [file ijerph-20-02598-s001.zip › ijerph-2120065-supplementary.pdf]

Supplementary material

Table S1: Odds ratios obtained from a logistic regression model predicting drop-out status (participating in all three wave vs. only in the first wave) by 2018 study variables.

**Drop-out predictor 2018**	**Odds ratio**		**95% CI**	**p-value**
Depression 2018 (ref: no)	0.82	0.64	1.05	0.1232
GAD 2018 (ref: no)	0.83	0.64	1.08	0.1775
Thoughts death self-harm 2018 (ref: no)	1.04	0.82	1.33	0.7307
ADHD 2018 (ref: no)	1.01	0.76	1.32	0.9710
RSOD 2018 (ref: less than monthly)				
Monthly	0.85	0.72	1.00	0.0549
Weekly	0.69	0.54	0.88	0.0035
Young women 2018 (ref: young men)	1.31	1.13	1.52	0.0003
Age 2018	1.01	0.96	1.06	0.7647
Non-Swiss citizen status 2018 (ref. Swiss citizen status)	0.50	0.41	0.60	0.0000
Household income 2018 (ref: < 6000.- CHF)				
about 6'000.- CHF	1.11	0.86	1.43	0.4231
> 6'000.- CHF	1.36	1.13	1.63	0.0013
Don't know	1.20	0.97	1.49	0.0967
Don't want to specify	0.90	0.69	1.17	0.4353
Ability to pay one's bills 2018 (ref: yes)				
No	0.65	0.52	0.81	0.0002
Don't know	0.86	0.66	1.11	0.2568
Language region 2018 (ref: German-speaking)				
French-speaking	0.99	0.84	1.17	0.9446
Italian-speaking	1.47	1.08	1.98	0.0129

ref = reference category. CI = Confidence interval. GAD = Generalized anxiety disorder. ADHD = Attention-deficit/Hyperactivity Disorder. RSOD = Risky single-occasion drinking.
